# Fear of Hunger: A Novel Eating Disorder Presentation in a 19-Year-Old Female from Gaza under Siege - A Case Study

**DOI:** 10.1192/j.eurpsy.2026.11383

**Published:** 2026-07-31

**Authors:** M. Abu-Slaih

**Affiliations:** Private clinic, Amman, Jordan

## Abstract

**Introduction:**

Eating disorders (EDs) in conflict-affected populations remain under-researched, with most literature focused on refugees outside active conflict zones. Current systems like DSM-5-TR and ICD-11 exclude eating disturbances caused by food scarcity, creating a diagnostic gap for cases arising from siege or famine. This case describes a trauma-related eating disturbance driven by fear of hunger and moral distress, falling outside.

**Objectives:**

To describe a unique restrictive eating disorder in a young woman living under siege in Gaza. The case highlights how extreme food insecurity and collective trauma can manifest as a distinct syndrome rooted in fear of starvation and survival guilt—rather than body-image distortion (as in AN) or sensory avoidance (as in ARFID). A secondary aim is to call for expanding diagnostic frameworks to include such trauma-related, context-bound disturbances.

**Methods:**

A 19-year-old female in Gaza was followed remotely during the 2023–2025 siege via online consultations and messages. Management included trauma-informed supportive psychotherapy and psychoeducation. Assessment relied on detailed interviews with the patient and her family and continuous monitoring of behavioral, cognitive, and physical changes.

**Results:**

The patient showed severe food restriction, ~10–12 kg weight loss, and behaviors like hiding or rationing food. Motivation stemmed not from body-image concerns but from intense fear of food depletion and guilt about eating while others starved. She expressed thoughts such as “If I eat now, there won’t be enough later,” and “It’s wrong to feel full when others are hungry.” No body-image distortion, weight-gain fear, or sensory aversion was observed. The pattern didn’t meet AN or ARFID criteria, as it reflected a realistic reaction to food scarcity. Treatment focused on validating her fears, addressing moral injury, and restoring structured eating, leading to partial nutritional and anxiety improvement, though core fears persisted.

**Conclusions:**

This case suggests a potential new diagnostic entity, *Famine-Related Food Avoidance*—a trauma-based eating disturbance distinct from known EDs. It exposes a major gap in DSM-5 and ICD-11, which fail to classify such psychologically devastating yet contextually understandable responses to imposed starvation. These presentations warrant clinical recognition and trauma-informed criteria that reflect the profound impact of siege and conflict on eating behavior and mental health.

**Disclosure of Interest:**

None Declared

